# Long term effects of micro-surgical testicular sperm extraction on androgen status in patients with non obstructive azoospermia

**DOI:** 10.1186/1471-2490-6-9

**Published:** 2006-03-20

**Authors:** Karel Everaert, Ilse De Croo, Wim Kerckhaert, Peter Dekuyper, Marc Dhont, Josiane Van der Elst, Petra De Sutter, Frank Comhaire, Ahmed Mahmoud, Nicolaas Lumen

**Affiliations:** 1 Department of Urology, Ghent University Hospital, De Pintelaan 185, B-9000 Ghent, Belgium; 2 Department of Gynaecology, Ghent University Hospital, De Pintelaan 185, B-9000 Ghent, Belgium; 3 Department of Endocrinology, Ghent University Hospital, De Pintelaan 185, B-9000 Ghent, Belgium

## Abstract

**Background:**

The aim of our study was to review the results of microsurgically performed testicular sperm extraction (TESE) and to evaluate its possible long term effects on serum testosterone (T).

**Methods:**

We operated on 48 men (35 +/- 8 years) with non-obstructive azoospermia (NOA). If no spermatozoa were found following a micro epididymal sperm extraction (Silber et al., 1994) and testicular biopsy, testicular microdissection was performed or multiple microsurgical testicular biopsies were taken. The mean follow-up of the serum T was 2.4 +/- 1.1 years.

**Results:**

Sperm was retrieved in 17/48 (35%) of the men. The per couple take home baby rate if sperm was retrieved was 4/17 (24%). Serum T decreased significantly at follow-up (p < 0.05) and 5/31 (16%) de novo androgen deficiencies developed

**Conclusion:**

In patients with non-obstructive azoospermia in whom no spermatozoa were found following a micro epididymal sperm aspiration and a simple testicular biopsy, we were able to retrieve spermatozoa in 35% of the men. The take home baby rate was 24% among couples with spermatozoa present upon TESE. *De novo *androgen deficiency occurred in 16% of the male patients following TESE indicating that, in men with NOA, long term hormonal follow up is recommended after TESE.

## Background

For men with non-obstructive azoospermia (NOA), retrieval of spermatozoa from the testes for assisted reproduction using intracytoplasmic sperm injection (ICSI) offers an opportunity for fertility despite limited sperm production [1-4]. The technique of testicular sperm extraction (TESE) via an open testicular biopsy was described for obstructive azoospermia by Schoysman et al. [5] as well as Craft et al. [6], and subsequently by Silber et al. [7] and Devroey et al. [1] for NOA.

Failure to extract spermatozoa may occur in up to 57% of TESE attempts [1-3,8,9]. Some authors have suggested a single large biopsy, others perform multiple smaller biopsies, and still others have reported excising a majority of the volume of the testis in an effort to sample enough tissue to extract spermatozoa from [10]. Multiple testicular biopsies can result in the loss of significant amounts of testicular tissue and can interrupt the testicular blood supply underneath the tunica albuginea with risks of testicular devascularization and subsequent atrophy of the testis [11].

In 1999 Schlegel et al. [12] reported on novel microsurgical techniques for TESE. They used an operating microscope to identify regions containing spermatozoa in the testes of men with NOA and performed multiple biopsies while avoiding the subtunical blood vessels. The technique is further referred to as microsurgical multiple testicular biopsies (Micro TESE). Subsequently, Schlegel et al. [12] achieved better retrieval rates using a microdissection technique (Microdissection TESE) while the amount of testicular tissue excised was reduced. The concept was simple: seminiferous tubules with Sertoli cells only (SCO) are thinner than tubules containing spermatogenic cells. The difference between the larger and smaller tubules is not visible without optical magnification. In Schlegel's hands this technique of microdissection resulted in an improvement of sperm retrieval rates from 45 to 63%. In men where no spermatozoa were found in a non-microsurgical single testicular biopsy procedure, the retrieval rate was 35% [12].

Concerning possible complications, many studies reported ultrasonographic evidence of residual testicular damage after TESE (reviewed in Schill et al. [13]). Schlegel and Su [11] reported complete testicular atrophy in two out of 64 patients following non-microsurgical TESE. Vascular damage can lead to a decrease of Leydig cell function causing a decrease of serum testosterone (T) level. Androgen deficiency may have serious long-term health consequences [14].

Manning et al. [15] described a decrease in serum T levels following non-microsurgical TESE in patients with NOA for up to one year after the procedure. Schill et al. [13] reported no decrease in serum T levels in patients with azoospermia after an average of 18 months following non-microsurgical TESE. However, the same study indicated that patients with NOA had lower serum T levels and that T response to human chorionic gonadotrophin (HCG) stimulation were more frequently impaired compared to men with obstructive azoospermia [16]. Therefore, they concluded that patients with NOA show an increased risk of androgen deficiency following non-microsurgical TESE and recommended long-term follow-up in this group of patients [16]. To date, there are few data on the long-term effects of TESE performed by microsurgery. One month after microdissection TESE, Hibi et al. [17] found no decrease in serum testosterone in 4 patients with NOA. Needle aspiration of testicular spermatozoa had no adverse effect on serum testosterone after up to nine months of follow-up [18].

So far, no serious adverse effects have been published following microsurgical TESE, qualifying these procedures as safe. Theoretically one should expect fewer complications compared to a classic testicular biopsy since better visualisation should lead to better safekeeping of the testicular end-arteries.

The aim of the study was to evaluate the outcome of micro TESE and microdissection TESE, and the possible long-term effects of these procedures on Leydig cell function as reflected by serum testosterone levels.

## Methods

### Study design

We retrospectively evaluated the outcome of microsurgical TESE in patients with NOA treated at the Ghent University Hospital, Belgium during the period January 1999 to June 2001. Only patients with a follow up of more then 1 year were included. Baseline data were available for all patients. Patients in whom postoperative hormonal data was missing were invited by mail to have a blood sample taken for the determination of serum hormones. The study was performed with the approval of the ethics committee of the Universtity Hospital of Ghent (Project EC UZG 2005/398).

### Patients

#### The male partner

During the period from January 1999 till June 2001, 48 patients with NOA treated by microsurgical TESE were identified in our hospital records. All men were examined pre-operatively according to the recommendations of the World Health Organization [19]. Azoospermia was proven in at least 2 ejaculates after centrifugation. Clinical postoperative evaluation was available in only 8 patients (39 patients were secondary referrals of whom 32 from abroad).

### Hormonal measurements

Commercial kits for radioimmunoassay were used to determine the serum concentrations of testosterone (Medgenics Diagnostics, Fleurus, Belgium); commercial kits for immuradiometric assays were used for determinations of LH and FSH (Medgenics Diagnostics). Intra- and interassay coefficients of variation for all these assays were below 10 and 15%, respectively.

Hormonal data were available on 45 patients at baseline. In March 2003 postoperative hormones levels were available from 31 out of the 45 patients. Patients with *de novo *androgen deficiency were asked to perform a second blood analysis. All blood samples were taken between 8 and 10 h 00 a.m.

In our laboratory, the minimal reference value for testosterone is 280 ng/dl. Biochemical androgen deficiency was diagnosed if testosterone levels in serum were below the reference value on 2 occasions.

### Surgical procedures

Surgery was performed on the day of the ovum pick-up in 23 patients while in 25 patients the previously retrieved frozen spermatozoa were used. Under general anaesthesia a median scrotal incision was performed and both testicles were exposed. Since we do not perform a pre-operative testicular biopsy, a micro epididymal sperm extraction was attempted in all patients together with a small single microsurgical testicular biopsy at the midline of the testis avoiding blood vessels [12]. If no spermatozoa were found, an operating microscope was used to obtain multiple biopsies (Micro TESE) if apparently normal seminiferous tubules were seen at the initial testicular biopsy (n = 31). With the use of an operating microscope, subtunical blood vessels are avoided as much as possible. If hairy or no seminiferous tubules were found [12] a microdissection TESE was performed (n = 17). The testes were incised and with the use of optical magnification, the larger tubules were excised, because of the greater chance of finding spermatogenic cells in these larger tubules.

Testicular histopathology was evaluated according to the method described by Johnsen [20] and was available for 46 out of 48 patients.

### The female Partner

The mean age of the female partners was 31 years with a standard deviation (SD) of 5 years. They were all treated with a short gonadotrophin-releasing hormone analogue protocol, followed by HCG. Oocytes were retrieved at our centre or in a transport setting [21], by transvaginal ultrasound-guided puncture of the ovarian follicles. ICSI was performed as previously described by our group [22] and 2 to 3 embryos were transferred.

Biochemical pregnancy was assessed by measuring serum HCG at least 15 days following embryo transfer on two successive occasions, and clinical pregnancy was ascertained by ultrasound at 6 weeks.

### Statistical analysis

Data are presented as mean +/- SD. Statistical analysis is performed using a Student t-test (paired when applicable), and Chi^2^-test (MedCalc, MedCalc Software, Mariakerke, Belgium).

## Results

A summary of the medical history, physical examination and hormone levels obtained at the initial evaluation of male partner is presented in Table 1. The mean follow-up period was 2.4 years (+/- 1.1 years).

### Complications of surgical procedures

Mild pain lasting 3–14 days was reported by all patients, while 2 patients complained of more severe pain lasting 1 month or more. Oral analgesics were needed during on average 6 days (rage 0–90 days). Surgery for haematoma was never needed. In 1 patient, unilateral cryptorchidism reoccurred after surgery and surgical revision of the wound was necessary in one patient with a spinal cord injury.

### Results of hormone determinations

Four out of 45 patients (8%) had serum testosterone levels below the reference range of our laboratory at the pre-operative evaluation (280 ng/dl).

Hormonal follow-up was available on 31 patients. A comparison between levels of serum hormones before TESE and at follow-up is presented in Table 2. Serum testosterone levels were on average 10 per cent lower at follow-up compared to pre-operative levels (p < 0.05). Five out of 31 patients (16%) were found to have a *de novo *androgen deficiency at follow-up. As these patients were foreigners no follow-up clinical data are available and patients were advised to seek medical help in their country. Among the 8 patients of whom a clinical evaluation was available during postoperative follow-up, no symptoms or signs of androgen deficiency were noted. They all also had normal testosterone levels both preoperatively and at the follow-up visit (433 +/- 99 ng/dl) (paired t-test: p > 0.05). No significant correlations were found between serum T and either age of the male partner or testicular volume (data not shown).

Table 2 also shows that serum levels of FSH and LH were slightly but not significantly increased at follow-up (p = 0.07, for both hormones).

### Sperm retrieval and conception rates

Spermatozoa were retrieved in 17 out of 48 patients (35.4%). 28 cycles of ICSI were performed in 17 couples with native spermatozoa present, and in 5 couples with donor spermatozoa of whom 2 cycles without embryo transfers. In September 2002, 53 embryo transfers were done resulting in 1 biochemical pregnancy, 1 miscarriage, 1 vanishing twin, 5 pregnancies and 4 babies (24%) among couples with spermatozoa present upon TESE. Fresh spermatozoa (n = 8 out of 25 men) resulted in 2 babies and frozen (n = 9 out of 23 men) in 2 babies.

## Discussion

To the best of our knowledge the first study this is reporting the long-term effects of microsurgical TESE in patients with NOA. Manning et al. [15] found that testosterone levels decreased initially after non-microsurgical TESE in most patients with partial recovery after 1 year. In our study only patients with a follow up of longer than 1 year were included. Other studied reported no decrease in serum testosterone [13,17,18]. Comparing our results to these studies is difficult due to the diverse techniques used to retrieve spermatozoa and different durations of post-operative follow-up.

Our results indicate that microsurgical TESE is associated with a significant long-term decrease of serum T levels. A relatively high incidence of T deficiency was recorded pre-operatively (8%) as well as after an average of 2.4 years of follow-up. It is generally agreed that testosterone decreases with age in the male [14]. However, it is unlikely that age *per se *is a major contributing factor to T deficiency in our patients since they were relatively young with a mean age of 34 years. Furthermore, T levels did not correlate significantly with age. On the other hand, patients with NOA are more prone to develop premature "andropause" due to the presence of testicular pathology, which effects are difficult to distinguish from those of TESE on serum T. As shown in Table 1, our patients had a total testicular volume which is on average at lower end of normality (30 ml) before surgery. So, Microsurgical TESE can be a reason of T deficiency but other factors, which can gradually compromise the Leydig cell function (e.g. cryptorchidism, testicular torsion, chemotherapy) should always be taken in consideration when the testosterone level declines. Further studies are needed to find the major contributing factor of long-term androgen deficiency after microsurgical TESE.

If one accepts that the observed decline in serum T is caused by TESE, several mechanisms may be responsible for the observed decline of serum T. These include loss of testicular tissue removed during surgery, surgical trauma and inflammation [11]. Also vascular injury to the testis can result from testicular surgery. Testicular blood supply penetrates the tunica albuginea and runs under the tunica before penetrating between the septa separating the seminiferous tubules. Therefore, these arteries are end-arteries[23]. Injury to these small arteries may cause devascularisation of a region of the testis. Schlegel and Su [11] reported that 82% of the patients who underwent a TESE procedure, had intratesticular abnormalities on ultrasound as long as 3 months afterwards. Most of the lesions seemed to disappear 6 months after TESE leaving only linear scars visible on ultrasound.

Patients need to be informed on the long-term consequences of TESE, including possible androgen deficiency and its therapy[13]. Before starting substitution therapy it seems advisable to wait for about 1 year after the surgery since some degree of spontaneous recovery may occur [15].

As our study is not a comparative evaluation of techniques (micro TESE vs. microdissection TESE), no conclusions can be drawn regarding which technique of microsurgery is to be preferred in the future. Anyhow, no significant differences in outcome and postoperative hormones were found.

Due to the adverse effect of TESE on serum T reported in this study, it is recommended that, whenever possible, an etiological treatment of the azoospermia should be attempted before resorting to TESE. For example, the treatment of varicocele and hypogonadal hypogonadism may result in the appearance of spermatozoa in the ejaculate and, in some cases, spontaneous pregnancy [16,24].

The take home baby rate of 24% among couples with spermatozoa present upon TESE is similar to the success rate of IVF or ICSI (25%) for other indications in our program [25]. This illustrates that the finding of sperm is the crucial step in patients with NOA, but once this step is successfully taken, the chances of getting normal fertilization, embryo development and pregnancy rates are the same as in any other couple.

## Conclusion

Among patients with NOA, in whom no spermatozoa are found following a micro-epididymal sperm aspiration and a simple testicular biopsy, we were able to retrieve spermatozoa in 35% by means of multiple microsurgical testis biopsies or testicular microdissection. The per couple take home baby rate was 24%. Long-term androgen deficiency is a potential risk of TESE and patients with NOA need to be informed about this possible complication.

## Competing interests

The author(s) declare that they have no competing interests.

## Authors' contributions

KE has drafted the manuscript, performed statistical analysis and literature review. He was one of the surgeons performing the operations. He performed the consultation of the infertile couple and included them in the study. He has given final approval of the version to be published.

IDC performed the anatomo-pathological analysis of the testicular biopsy.

WK participated in drafting the manuscript and in performing the statistical analysis. He was one of the surgeons performing the operations.

PDK participated in drafting the manuscript and performed literature review. He was one of the surgeons performing the operations.

MD performed statistical analysis of the female partner and the final conception rate. He performed the consultation of the female partner

JVDE performed the analysis of the sperm obtained by surgery. She performed a critical review of the text.

PDS performed the retrieval of the oocytes of the female partner and performed IVF/ICSI. She performed a critical review of the text. She performed literature review about ICSI.

FC performed a critical review of the text and performed literature review about azoospermia

AM performed analysis of the sperm before operation. He also did the statistics and participated in writing the   article.

NL performed acquisition of data in the medical files. He participated in drafting the manuscript. He was one of the surgeons performing the operations. He has given final approval of the version to be published.

## Pre-publication history

The pre-publication history for this paper can be accessed here:



## References

[B1] Devroey P, Liu J, Nagy Z, Goossens A, Tournaye H, Camus M, Van Steirteghem A, Silber S (1995). Pregnancies after testicular sperm extraction and intracytoplasmic sperm injection in non-obstructive azoospermia. Human Reproduction.

[B2] Kahraman S, Ozgur S, Alatas C, Aksoy S, Tasdemir M, Nuhoglu A, Tasdemir I, Balaban B, Biberoglu K, Schoysman R, Nijs M, Vanderzwalmen P (1996). Testicular sperm extraction and intracytoplasmic sperm injection in non-obstructive azoospermic men. Human Reproduction.

[B3] Schlegel P, Palermo G, Goldstein M, Menedez S, Zaninovic N, Veeck L, Rosenwaks Z (1997). Testicular sperm extraction with intracytoplasmic sperm injection for non-obstructive azoospermia. Urology.

[B4] Jow W, Steckel J, Schlegel PN, Magid MS, Goldstein M (1993). Motile sperm in human testis biopsy specimens. Journal of Andrology.

[B5] Schoysman R, Vanderzwalmen P, Nijs M, Segal-Bertin C, Geerts L, Van de Casseye M (1993). Successful fertilization by testicular spermatozoa in an in-vitro fertilization programme. Human Repoduction.

[B6] Craft I, Bennett V, Nicholson N (1993). Fertilizing ability of testicular spermatozoa. Lancet.

[B7] Silber S, Nagy Z, Liu J (1995). The use of epididymal and testicular spermatozoa for intracytoplasmic sperm injection: the genetic implications for male infertility. Human Reproduction.

[B8] Friedler S, Raziel A, Strassburger D, Soffer Y, Komarovsky D, Ron-El R (1997). Testicular sperm retrieval by percutaneous fine needle sperm aspiration compared with testicular sperm extraction by open biopsy in men with non-obstructive azoospermia. Human Reproduction.

[B9] Rosenlund B, Kvist U, Ploen L, Rozell BL, Sjoblom P, Hillenso T (1998). A comparison between open and percutaneous needle biopsies in men with azoospermia. Human Reproduction.

[B10] Silber S, Nagy Z, Devroey P, Tournaye H, Van Steirteghem AC (1997). Distribution of spermatogenesis in the testicles of azoospermic men: the presence or absence of spermatids in the testis of men with germinal failure. Human Reproduction.

[B11] Schlegel PN, Su L (1997). Physiologic consequences of testicular sperm extraction. Human Reproduction.

[B12] Schlegel P (1999). Testicular sperm extraction: microdissection improves sperm yield with minimal tissue excision. Human Reproduction.

[B13] Schill T, Bals-Pratsch M, Kupker W, Sandmann J, Johannison R, Diederich K (2003). Clinical and endocrine follow-up of patients after testicular sperm extraction. Fertility and Sterility.

[B14] Comhaire FH (2000). Andropause: hormone replacement therapy in the ageing male. European Urology.

[B15] Manning M, Junemann K, Alken P (1998). Decrease in testosterone blood concentrations after testicular sperm extraction for intracytoplasmic sperm injection in azoospermic men. Lancet.

[B16] Bouloux P, Warne D, Loumaye E (2002). FSH Study Group in Men's Infertility. Efficacy and safety of recombinant human follicle-stimulating hormone in men with isolated hypogonadotropic hypogonadism. Fertility and Sterility.

[B17] Hibi H, Taki T, Yamada Y, Honda N, Fukatsu H, Yamamoto M, Asada Y (2002). Testicular sperm extraction using microdissection for non-obstructive azoospermia. Reproductive medicine and Biology.

[B18] Westlander G, Ekerhovd E, Granberg S, Lycke N, Nilsson L, Werner C, Bergh C (2001). Serial ultrasonography, hormonal profile and antisperm antibody response after testicular sperm aspiration. Human Reproduction.

[B19] Rowe PJ, Comhaire FH, Hargreave TB, Mahmoud AM (2000). World Health Organization manual for the standardized investigation, diagnosis and management of the infertile male.

[B20] Johnsen SG (1970). Testicular biopsy score count – a method for registration of spermatogenesis in human testes: normal values and results in 335 hypogonadal males. Hormones.

[B21] Coetsier T, Verhoeff A, De Sutter P, Roest J, Dhont M (1997). Transport IVF/ICSI: a prospective randomised study. Human Reproduction.

[B22] Dozortsev D, De Sutter P, Rybouchkin A, Dhont M (1996). Methodology of intracytoplasmic sperm injection in the human. Assisted Reproduction Review.

[B23] Schlegel P, Chang T, Walsh P, Retik A, Stamey T and Vaughan E (1991). The testis, epididymis and ductus deferens. Campbell's Urology.

[B24] Matthews G, Matthews E, Goldstein M (1998). Induction of spermatogenesis and achievement of pregnancy after microsurgical varicelectomy in men with azoospermia and severe oligoasthenospermia. Fertility and Sterility.

[B25] De Sutter P, Van der Elst J, Coetsier T, Dhont M (2003). Single embryo transfer and multiple pregnancy rate reduction in IVF/ICSI: a 5 year appraisal. Reproductive Biomedicine Online.

